# Yolk Fatty Acid Profile and Amino Acid Composition in Eggs from Hens Supplemented with ß-Hydroxy-ß-Methylbutyrate

**DOI:** 10.3390/foods12203733

**Published:** 2023-10-11

**Authors:** Aleksandra Dajnowska, Ewa Tomaszewska, Sylwester Świątkiewicz, Anna Arczewska-Włosek, Piotr Dobrowolski, Piotr Domaradzki, Halyna Rudyk, Oksana Brezvyn, Viktor Muzyka, Ihor Kotsyumbas, Marcin B. Arciszewski, Siemowit Muszyński

**Affiliations:** 1 Department of Animal Anatomy and Histology, Faculty of Veterinary Medicine, University of Life Sciences in Lublin, 20-950 Lublin, Poland; aleksandra.dajnowska@up.lublin.pl (A.D.); mb.arciszewski@wp.pl (M.B.A.); 2 Department of Animal Physiology, Faculty of Veterinary Medicine, University of Life Sciences in Lublin, 20-950 Lublin, Poland; 3 Department of Animal Nutrition and Feed Science, National Research Institute of Animal Production, 32-083 Balice, Poland; sylwester.swiatkiewicz@iz.edu.pl (S.Ś.); anna.arczewska@iz.edu.pl (A.A.-W.); 4 Department of Functional Anatomy and Cytobiology, Faculty of Biology and Biotechnology, Maria Curie-Sklodowska University, 20-033 Lublin, Poland; piotr.dobrowolski@umcs.lublin.pl; 5 Department of Quality Assessment and Processing of Animal Products, Faculty of Animal Sciences and Bioeconomy, University of Life Sciences in Lublin, 20-950 Lublin, Poland; piotr.domaradzki@up.lublin.pl; 6 State Scientific Research Control Institute of Veterinary Medicinal Products and Feed Additives, 79000 Lviv, Ukraine; galusik.77@gmail.com (H.R.); brezvun@gmail.com (O.B.); muzyka@scivp.lviv.ua (V.M.); ihork@scivp.lviv.ua (I.K.); 7 Department of Biophysics, Faculty of Environmental Biology, University of Life Sciences in Lublin, 20-950 Lublin, Poland; siemowit.muszynski@up.lublin.pl

**Keywords:** egg quality, lipids, n-3 PUFA, supplementation

## Abstract

In recent years, a supplementation of livestock animals, including poultry, with β-Hydroxy-β-methylbutyrate (HMB) has gained attention for its effects on protein and fat metabolism. This study investigates the effects of HMB in the laying hen diet on egg quality, focusing on amino acid and fatty acid composition. Laying hens were supplemented with 0.02% HMB, with performance parameters and egg components analyzed. HMB supplementation led to increased albumen weight, influencing egg weight while also reducing feed intake per egg without affecting laying rate, yolk indices, fat, or cholesterol content. Notably, the study revealed significant changes in egg amino acid and fatty acid profiles due to HMB supplementation. Various amino acids, including glycine, serine, and isoleucine, were altered in the yolk, impacting nutritional value and potential health benefits. Regarding fatty acids, the study observed changes in both saturated as well as n-6 and n-3 fatty acids, affecting the overall lipid profile of egg yolks. However, the shifts in fatty acid composition could have implications for cardiovascular health due to altered ratios of n-6/n-3 fatty acids. Further research is required to comprehensively understand the implications of these findings for consumer-oriented egg quality and health benefits.

## 1. Introduction

The consumption of high-caloric, fat-rich processed food increased last time and has led to a rise in chronic diseases like hypertension, obesity, cardiovascular diseases, atherosclerosis, type 2 diabetes, and certain forms of cancer [1]. On the other hand, a healthy diet includes an adequate amount of proteins and crucial macronutrients that play a pivotal role in building and repairing tissues, supporting immune function, producing enzymes and hormones, and helping maintain muscle mass. It is essential to choose a variety of protein-rich foods to ensure a well-balanced diet. However, a healthy diet is not solely centered around protein; it should also include a balance of fats. A healthy diet includes moderate fat, focusing on n-3 polyunsaturated fatty acids (PUFA), and limiting n-6 PUFA [1].

Eggs are recognized as a cost-effective source of high-quality protein [2]. They offer a complete meal with essential amino acids, making them accessible to people across different economic backgrounds [3]. The demand for protein-rich foods with additional health benefits is rising. Eggs’ amino acid profile can affect their taste [4]. There is also growing consumer interest in the lipid content of egg yolks and its impact on health [5,6].

The albumen is a valuable source of protein, while the yolk is rich in lipids, which serve as an efficient source of energy. Fatty acids (FA), present in their composition, are also an important source of biologically and nutritionally important substances. The composition of egg yolk lipids, aside from FA, includes triglycerides, mono- and diglycerides, cholesterol, cholesterol esters, and phospholipids [7].

Egg chemical composition can be influenced by factors such as age, the genes of the hens, or the housing system [8,9]. The specific composition of their feed also affects egg composition [10,11,12,13]. Specifically, the FA profile can be modified. To achieve this, the diets of hens are often enriched with oils of different sources (fish, vegetables, herbs, oilseeds, and algae) [14,15,16]. 

Beta-hydroxy-beta-methylbutyrate (HMB) is a naturally occurring five-carbon organic acid produced primarily in the cytosol of the liver and muscle cells [17] through the metabolic breakdown/oxidation of leucine. It is further converted through beta-hydroxy-beta-methylglutaryl-CoA into mevalonate and sterols, acetoacetate and beta-hydroxybutyrate, and acetylo-CoA [18]. HMB is involved in regulating muscle protein synthesis. HMB has been shown to prevent the degradation of muscle proteins and reduce muscle damage and inflammation. HMB exhibits immunomodulatory effects, supporting immunity and reducing infection risk [19,20,21]. Studies on pigs supplemented with HMB have shown that it regulates lipid metabolism, promoting fat oxidation, which could potentially improve body composition and aid in fat loss [22,23,24]. Additionally, HMB enhanced piglets bone health by promoting bone formation, reducing resorption, and increasing bone density [25,26,27,28].

Given the multiple benefits of HMB as a nutritional supplement influencing protein and fat metabolic processes, HMB has recently been extensively studied as a food additive in poultry nutrition, including layers [22,23,24,29]. In this study, we tested the hypothesis that supplementing the diet of laying hens with HMB, due to its effects on the hens’ protein and fat metabolism, could influence egg quality. Specifically, this could lead to alterations in amino acid and fatty acid composition, thereby potentially enhancing the nutritional value and health benefits of eggs for consumers. Our research objectives, chosen to meet these goals, primarily focused on amino acid and fatty acid analyses but also included basic performance metrics for layers and fundamental albumen and yolk indices. We concentrated on hens supplemented with HMB during their post-peak laying period (31–60 weeks of life), a phase when egg quality typically tends to decline due to age-related changes in the hen’s physiology.

## 2. Materials and Methods

### 2.1. Birds and Experimental Diets

The study employed 48 Bovans Brown laying hens procured from a local supplier. Upon arrival, hens were kept in cages accommodating two birds per cage, in compliance with the established housing regulations for research involving farm animals in the field of agriculture (cage dimensions of 30 × 120 × 50 cm, w × d × h, ensuring 1800 cm^2^ of floor space per hen). The experimental timeline consisted of a pre-experimental phase (age weeks 18–30), during which all birds were provided with the standard soybean-wheat-corn-based commercial pre-laying and laying diet. At the commencement of the experimental phase (age weeks 31–60), the birds were randomly allocated to either the control group or the group receiving dietary supplementation with ß-Hydroxy-ß-methylbutyrate (the HMB group). Each group was composed of *n* = 12 replicate cages with two hens per cage. The dietary composition was based on a soybean-wheat-corn mixture that adhered to the nutrient requirements outlined in the recommended standards for laying hens [30]. The basal diet given to the hens in the HMB group was enriched with 0.02% HMB (OstroVit, Zambrów, Polska). The selection of the 0.02% HMB dosage was guided by the existing literature on HMB supplementation in broiler chickens, even though this specific dose had not been previously studied. Nissen et al. [31] explored dosages of 0.003%, 0.01%, 0.03%, 0.05%, and 0.09% over a 7-week period. Peterson et al. [32,33] examined the effects on broilers supplemented with doses of 0.01%, 0.05%, 0.075%, and 0.1% for 3 weeks. Buyse et al. [34] utilized a dosage of 0.03% for 5.5 weeks. Qiao et al. [35] evaluated dosages of 0.05% and 0.1% over a 6-week period, while Wan et al. [29] researched HMB at doses of 0.05%, 0.1%, and 0.15%. 

The feed consumption (g/hen/day), egg laying rate (eggs number/hen/day), average egg weight (g), and feed conversion rate (g feed/g eggs) were monitored per replicate cage throughout the entire experimental period. 

### 2.2. Sample Preparation and Analysis

During the last two days of the supplementation period, marking the end of the 30th of the experimental week, from eggs collected from each replicate cage, two eggs were randomly selected (*n* = 24 eggs per group) and analyzed. After breaking an egg onto a glass plate, the albumen height was measured with an electronic tripod-mounted height gauge (QDC Egg Quality System, Technical Services and Supplies, York, UK). Next, to determine the proportions of yolk and albumen, their weights were recorded. For the albumen, the Haugh unit was calculated [36].

The egg yolk and albumen were analyzed according to AOAC International methods for moisture content (oven drying method 934.01), crude fat (ether extract method 920.390), protein content (using the Kjeldahl system and conversion 6.25 × P to crude protein, method 954.01), and ash content (gravimetric method 942.05) [37].

The determination of the AA profile of egg albumen, excluding cystine, methionine, and tryptophan, was carried out following AOAC method 994.12 using the automatic AA analyzer (INGOS AAA400, Ingos Corp., Prague, Czech Republic). Methionine (as methionine sulphone) and cystine (as cysteic acid) were analyzed after performing acid oxidation [38]. Tryptophan content was determined using the ion exchange chromatography method [39].

Yolk lipids were extracted using the Folch method [40]. The measurement of yolk cholesterol content was performed using a Varian 3400 gas chromatograph (Varian Inc., Walnut Creek, CA, USA), using 5-alpha-cholestan as an internal standard [41]. 

The analysis of FA composition was performed as previously described [42]. Briefly, extracted fatty acid methyl esters (FAMEs) were processed using method Ce 2–66 of AOAC [37] with gas chromatography (CG 399, Varian, Walnut Creek, CA, USA). For FAME identification and quantification, retention times were compared to reference mixtures (Supelco Inc., Bellefonte, PA, USA; Larodan AB, Solna, Sweden). The percentage fraction of each FA, as % of total FA, was calculated for each FAME using the FA-specific Sheppard factor [37]. The following sums of fatty acids were calculated: saturated FA (Σ SFA), mono-unsaturated FA (Σ MUFA), polyunsaturated FA (Σ PUFA), n-3 PUFA (Σ n-3 PUFA), n-6 PUFA (Σ n-6 PUFA), long chain PUFA (LC-PUFA), n-3 LC-PUFA (Σ n-3 PUFA), n-6 LC-PUFA (Σ n-6 PUFA).

Additionally, health lipid indices were calculated based on the formulas given in [43]: saturation index (S/P), nutritional value (NV), atherogenicity index (AI), thrombogenic index (TI), and the h/H ratio. 

### 2.3. Statistical Analyses

The data are presented as means ± SEM. All statistical procedures were conducted using Statistica software (v. 13.3, TIBCO Software Inc., Palo Alto, CA, USA). The values of each trait measured in individual eggs were averaged per cage before undergoing statistical analysis. The replicated cage was considered the experimental unit (*n* = 12 per group). The sample size analysis (employing a two-tailed Student’s *t*-test with α = 0.05 and power = 0.8) indicated that a sample size of *n* = 12 is adequate to detect changes ranging from 10–19% in AA values in egg albumen [44]. 

The data were checked for normality (the Shapiro-Wilk test) and homogeneity of variances (the Levene test). Data with a normal distribution were analyzed using a two-tailed Student’s *t*-test. When data demonstrated unequal variances, Welch’s correction was applied. For data not conforming to a normal distribution, the Mann-Whitney U test was employed. A significance level of *p* < 0.05 was set for all analyses.

For the calculated partial sum of FA, principal component analysis (PCA) was intended. However, Bartlett’s test of sphericity and the Kaiser–Meyer–Olkin (KMO) measure used to assess the suitability of the PCA indicated that the data did not meet the assumptions for PCA. Therefore, PCA was not carried out.

## 3. Results

Taking into account the whole laying period, HMB supplementation did not influence the laying rate but increased total mass and daily mass of eggs, decreasing total feed intake and daily feed intake per gram of egg (Table 1). When evaluating the egg’s weight specifically at the end of the experimental period, it was shown that the rise in egg mass was primarily due to an increase in albumen weight (Table 2), which in turn was characterized by an elevated protein content (Table 3). In yolk, HMB supplementation resulted in a decrease in ash content. When assessing AA content in albumen, the increase of asparagine and isoleucine and the decrease of cysteine and lysine were noted (Table 4). In the yolk, there was a decrease in the content of glycine, histidine, proline, serine, and valine and an increase in isoleucine content following HMB supplementation in hens (Table 5).

Analysis of the egg yolk FA composition revealed that HMB supplementation increased the levels of C16:0 palmitic acid (*p* < 0.01), leading to a rise in total SFA content (*p* < 0.01). On the other hand, there was a reduction in C17:0 margaric acid (*p* < 0.01), which brought down the total OCFA content (*p* < 0.05). Regarding monounsaturated fatty acids, there was an uptick in 16:1n-7 palmitoleic acid (*p* < 0.05) and C18:1c11 vaccenic acid (*p* < 0.01), contributing to an overall increase in total cis MUFA (*p* < 0.01). For polyunsaturated fatty acids, decreases were observed in C18:2n-6 linoleic acid (*p* < 0.001), C18:3n-3 linolenic acid (*p* < 0.001), and C22:6n-3 docosahexaenoic acid (DHA, *p* < 0.001). This led to a significant drop in the total PUFA content (*p* < 0.001) and all its subfractions (PUFA n-6, PUFA n-3, long-chain PUFA, and LC n-3, *p* < 0.001 for all). These alterations influenced FA ratios, raising the PUFA n-6/n-3 ratio (*p* < 0.01) and reducing the PUFA/SFA ratio (*p* < 0.001) (Table 6). As a result, alterations in the identified health lipid indices were observed. There was an increase in all indices (S/P, TI, AI, and NV), while the h/H ratio decreased in the HMB group (Table 6). 

## 4. Discussion

Although egg proteins are distributed in albumen and yolk, albumen proteins are renowned for their functional attributes and are extensively employed as ingredients to improve the texture and flavor of a diverse range of food items. Furthermore, albumen proteins hold the potential to serve as a reservoir of bioactive proteins and peptides. These bioactive components derived from egg proteins can contribute to health-enhancing effects that go beyond their recognized nutritional significance. For this reason, eggs are considered a valuable nutrient source for humans [45]. In human nutrition, essential AA include arginine, leucine, isoleucine, lysine, methionine, phenylalanine, threonine, tryptophan, and valine [46]. Our research revealed that the most prevalent amino acids in egg albumen were aspartic acid, serine, glutamic acid, valine, leucine, and lysine, with histidine being the most limited AA. These findings align with prior studies, which found similar AA profiles across various egg sources [3]. 

Albumen quality often depends on protein secretions, which are more or less dependent on the dietary protein composition and particular AA intake. In this study, HMB supplementation had diverse effects on amino acid composition, impacting both albumen and yolk quality. The inclusion of HMB in the hens’ diet resulted in increased levels of specific amino acids, notably asparagine in albumen and isoleucine in both albumen and yolk. These amino acids have various physiological roles. Asparagine is vital for brain development and overall protein synthesis [47], while isoleucine, a branched-chain amino acid, is known for its anabolic properties and therapeutic benefits in conditions like chronic renal failure, sepsis, and trauma [48].

Conversely, HMB supplementation led to decreased levels of certain amino acids. Cysteine and lysine levels in albumen were reduced, along with glycine, histidine, proline, and serine levels in yolk. Cysteine plays a significant role in energy supply and antioxidant functions, while lysine is essential for protein synthesis and cell growth [49]. Glycine serves as a building block for biomolecules and has anti-inflammatory properties, and proline is crucial for collagen formation [50]. Histidine participates in deamination and histamine production, which are important for inflammation [51]. Serine is a precursor for various compounds and is vital for metabolic processes [52].

These alterations in amino acid composition not only affect the quality and nutritional value of albumen but may also influence the taste of the eggs. Different amino acids contribute to taste perceptions such as sweetness, sourness, saltiness, bitterness, and umami. For example, umami taste enhancers include glutamic acid and aspartic acid, while sweet taste contributors encompass amino acids like methionine, glycine, and lysine [4]. 

HMB is not directly involved in amino acid synthesis; however, understanding the impact of HMB supplementation on egg quality is important. HMB supplementation may influence the availability of specific amino acids in the hens’ bodies. It is possible that HMB could affect the synthesis of specific amino acids or the incorporation of these amino acids into egg proteins. The composition of eggs is influenced by the diet and overall health of laying hens. If HMB supplementation leads to changes in the hens’ muscle protein synthesis and amino acid metabolism, it could indirectly affect the composition of eggs, including the levels of specific amino acids and proteins in the eggs. Research specific to this topic would be needed to provide more detailed insights into these relationships. Specific molecular and cellular pathways related to HMB metabolism and its effects on muscle protein synthesis and egg composition would need to be investigated to provide a comprehensive understanding of the mechanisms involved.

Employing nutritional approaches to enhance egg quality, particularly the FA profile, is a recognized and extensively explored strategy [53]. Manipulating the feed formulation can alter the FA composition of egg yolks, resulting in the production of eggs enriched with PUFA, offering advantages beyond fundamental nutritional aspects [54,55]. Derived from the standardized poultry diet, approximately 30–35% of the total FA consists of SFA, 40–45% of MUFA, and 20–25% of PUFA. Yolk lipids are also a source of LC-PUFA [56].

It is difficult to compare the results obtained from the current study with the data presented in the literature since there are no available reports on HMB effects on egg yolk FA profiles in laying hens except for one very recent study showing the HMB effect on FA profiles in the meat of broiler chickens [29]. The study demonstrated that 0.10% and 0.15% HMB supplements reduced C18:1c9n MUFA levels and increased total SFA content. Additionally, these supplements raised MUFA and lowered PUFA content in leg muscle, resulting in a decreased PUFA-to-SFA ratio. This aligns with findings in studies on animals other than poultry, suggesting the potential for nutritional modifications of fatty acids in animal products [57,58,59,60]. Changes in the muscle fatty acid metabolism might extend to affect the fatty acid profiles of eggs, although this has not been directly confirmed in either of those studies. While there are not specific studies on egg fatty acids after HMB supplementation, it is worth noting that insights from research on fatty acids in meat could provide valuable context for understanding potential implications on general fatty acid metabolism and their profile in egg yolk. 

On the other hand, dietary 0.05% HMB decreased both essential and non-essential amino acid contents, while 0.15% HMB specifically decreased non-essential amino acids. The supplementation of HMB, particularly at 0.05% and 0.15% levels, resulted in decreased nutritional values in meat and reduced lipogenesis in leg muscles [29]. It is important to emphasize once more that the prior study focused on analyzing FA in muscles, making the current presentation unique in its approach. The present study revealed that the addition of HMB did not impact the overall lipid and cholesterol content of the egg yolk, aligning with previously documented levels [61]. The increase in palmitic acid contradicts the expected cardiovascular benefits of HMB supplementation, while a reduction in OCFA like pentadecanoic acid (C15:0) and heptadecanoic acid (C17:0) may not favor cardiovascular health [62].

We observed an increase in two MUFAs, palmitoleic acid (C16:1c9) and cis-vaccenic acid (C18:1c11). While the total MUFA content also rose, our data show lower levels compared to previous research [62,63]. MUFA-rich diets, particularly those with cis-vaccenic acid, have been linked to positive health outcomes, including improved cholesterol levels and potential hypotensive effects. Palmitoleic acid, an omega-7 fatty acid, has anti-inflammatory and insulin-sensitizing properties [64]. This elevation in MUFA within egg yolks after HMB supplementation may benefit consumers.

Egg yolks contain essential PUFA, including linoleic acid (C18:2n-6, LA), α-linolenic acid (C18:3n-3, ALA), eicosapentaenoic acid (C20:5n-3, EPA), and docosahexaenoic acid (C22:6n-3, DHA). In our study, we found substantial PUFA in egg yolks. However, HMB supplementation led to a significant decline in the proportions of total, n-6, and n-3 PUFA, mainly due to reductions in LA, ALA, and DHA. LA is crucial because it serves as a precursor for longer-chained PUFA, such as arachidonic acid (C20:4n-6, ARA) [65]. Notably, essential PUFA like dihomo-γ-linolenic acid (C20:3n-6, DGLA) and docosapentaenoic acid (C22:5n-6, DPA) were unaffected by HMB supplementation. DGLA has anti-inflammatory and anti-thrombotic effects. Although GLA has pro-inflammatory properties, its conversion to DGLA contributes to the production of anti-inflammatory prostaglandins and thromboxane. Moreover, DGLA is associated with an antithrombotic effect. N-6 FAs have shown potential in treating diabetic neuropathy, rheumatoid arthritis, and pain reduction [66,67].

Omega-3 fatty acids are valued for their anti-inflammatory properties and potential to prevent conditions like heart disease and dementia. These essential fatty acids are vital for a healthy nervous system and must be obtained through diet. In this study, yolk’s n-3 PUFA composition decreased after HMB supplementation, with significant reductions in alpha-linolenic acid (C18:3n-3, ALA) and docosahexaenoic acid (C22:6n-3, DHA). ALA is a fundamental precursor for other omega-3 fatty acids, while DHA is crucial for brain and retinal health [68]. Docosapentaenoic acid (C22:5n-3, DPA), involved in the inflammatory response, remained unchanged. LC-PUFA, including both the n-3 and n-6 families, decreased in total content in egg yolks in this study.

In this study, HMB supplementation in hens reduced both n-3 and n-6 PUFA in egg yolks, leading to an increased n-6/n-3 PUFA ratio. A balanced intake of these PUFA types is crucial, as they compete for the same enzymes while serving distinct biological functions [69]. The imbalanced n-6/n-3 ratio, characterized by low n-3 LC-PUFA consumption and high n-6 PUFA intake, affects factors like lipid metabolism, inflammation, oxidative stress, and endothelial function, all of which contribute to cardiovascular disease development. Maintaining a lower n-6/n-3 PUFA ratio has been linked to favorable cardiovascular effects [70].

The ratios of PUFA/SFA and n-6/n-3 are frequently employed to evaluate the nutritional quality of fats. Dietary ratios exceeding 0.45 for PUFA to SFA and staying below 4 for n-6 to n-3 are generally regarded as suitable to provide protection against the onset of ischemic heart disease [71]. The n-6/n-3 ratio within the yolks of our hens, regardless of HMB supplementation, exceeded recommended levels. This observation might suggest that factors such as the genetics of the hens played a role in influencing this ratio. While the PUFA/SFA ratio exceeded 0.45 in the control group yolks, a lower value was observed after HMB supplementation. In comparison to literature values (31.37%), the content of palmitic acid, the most prevalent SFA, was relatively lower (24.4–25.6%) in our hens’ egg yolks. Furthermore, our study recorded a lower overall SFA content (31.77–33.104%) in contrast to the data reported by [72] (44.8%). In a separate study, higher SFA concentrations (myristic—0.69%; palmitic—27.53%; stearic—11.61%), lower MUFA concentrations (39.98%; oleic acid—36.78%), and elevated PUFA concentrations (22.95%; linoleic acid—21.64%) were revealed [62] compared to our study. Similarly, another study reported varying concentrations of fatty acids, including myristic (0.29%), palmitic (22.46%), stearic (6.17%), oleic (40.53%), linoleic (20.6%), ALA (0.7%), GLA (0.05%), and AA (1.40%) [63]. 

The existing literature presents a plethora of conflicting findings concerning the FA composition of yolks. Discrepancies in the deposition of DHA and EPA across different poultry species, such as chicken, duck, turkey, and geese, are well-established, regardless of diet or feed variations. Chicken egg yolk, for instance, contains a higher DHA content compared to other poultry species [73]. Additionally, the FA profile can be altered by dietary factors [14]. Nonetheless, in the yolk of hens, regardless of HMB supplementation, some FAs like palmitic acid (SFA, C16:0), oleic acid (MUFA, C18:1n-9), and LA (PUFA, C18:2n-6), emerged as the most abundant fatty acids, and these findings align with similar studies [74,75]. Furthermore, the supplementation of HMB to our hens resulted in increased AI, NV, TI, and S/P indices while concurrently decreasing the h/H index. These changes indicate a decline in the overall nutritional quality of the egg yolks, as observed in a study by Chen and Liu [43]. These findings were somewhat unexpected, as typically, fats with lower TI and AI indices and a higher h/H index are associated with better nutritional quality. 

All these changes in the fatty acid profile in the yolk of laying hens supplemented with HMB could be a probable result of an alteration of the hen’s metabolism. HMB is converted to β-hydroxy-β-methylglutaryl-coenzyme A (HMG-CoA), which serves to form fatty acids in a complex process involving various enzymatic reactions. Firstly, HMG-CoA is converted to acetyl coenzyme A (acetyl-CoA) by HMG-CoA lyase, which can be carboxylated to form malonyl coenzyme A (malonyl-CoA) through the action of acetyl-CoA carboxylase, which requires biotin and ATP. Malonyl-CoA serves as the main extender unit for fatty acid synthesis and is transferred to the acyl carrier protein (ACP) via malonyl-CoA:ACP transacylase. Acetyl-CoA can also be transferred to ACP through acetyl-CoA:ACP transacylase. The fatty acid synthase (FAS) complex plays a crucial role in the condensation of acetyl-CoA and malonyl-CoA, forming a 4-carbon unit. This unit undergoes a series of reduction, dehydration, and further reduction reactions to produce a saturated 4-carbon fatty acid. The FAS complex can repeat this cycle by adding more malonyl-CoA units to the growing fatty acid chain until it reaches palmitoyl-CoA, a 16-carbon molecule. Palmitoyl-CoA can be further elongated or desaturated by other enzymes to yield various types of fatty acids [76]. It is crucial to understand that HMG-CoA is a critical component in the process of cholesterol synthesis. Since cholesterol and fatty acids are interconnected in various metabolic pathways, any disruption in the synthesis of one may affect the other. Thus, disturbances in HMG-CoA availability could potentially impact fatty acid synthesis in the yolk [77].

## 5. Conclusions

The presented investigation demonstrated that supplementing laying hens with HMB at a 0.02% dosage led to substantial alterations in the amino acid and fatty acid composition of eggs. These modifications may have implications for the quality of eggs as a food product. While our findings provide valuable insights, additional research is warranted to comprehensively assess the consumer-oriented advantages stemming from these changes in egg composition.

## Figures and Tables

**Table 1:** The effect of dietary HMB supplementation on basic performance metrics.

Item	Control	HMB	*p*-Value
Total number of eggs	1175 ± 4	1173 ± 3	0.683
Total mass of eggs, kg	69.97 ± 0.19	72.16 ± 0. 322	<0.001
Laying rate, %	96.43 ± 0.23	96.3 ± 0.27	0.718
Daily mass of eggs, g/hen/day	55.22 ± 0.17	57.26 ± 0.39	<0.001
Feed consumption, g/hen/day	144.97 ± 0.85	143.31 ± 0.72	0.153
Daily feed intake per egg, g	119.45 ± 0.60	117.65 ± 0.59	0.044
Feed intake per egg, g	124.79 ± 0.50	122.27 ± 0.48	0.002
Feed conversion rate per egg, g/g	2.06 ± 0.10	1.99 ± 0.09	0.476

The data are presented as means ± SEM (*n* = 12). For details on the *p*-value calculation procedures, please refer to Section 2 under Section 2.3.

**Table 2:** Albumen and yolk indices following 30 weeks of HMB supplementation.

Item	Control	HMB	*p*-Value
Egg weight, g	59.47 ± 0.87	63.13 ± 1.12	0.026
Yolk weight, g	16.8 ± 0.48	16.87 ± 0.40	0.916
Albumen weight, g	35.33 ± 0.83	38.29 ± 0.72	0.013
Yolk fraction, %	28.25 ± 0.70	26.71 ± 0.37	0.068
Albumen fraction, %	59.36 ± 0.84	60.67 ± 0.49	0.191
Albumen height, mm	5.93 ± 0.25	6.19 ± 0.27	0.491
Haugh units	75.9 ± 1.83	76.41 ± 2.12	0.795

The data are presented as means ± SEM (*n* = 12). For details on the *p*-value calculation procedures, please refer to Section 2 under Section 2.3.

**Table 3:** Albumen and yolk composition following 30 weeks of HMB supplementation.

Item	Control	HMB	*p*-Value
Albumen Components			
Water, %	88.140 ± 0.307	88.622 ± 0.111	0.161
Protein, %	8.652 ± 0.305	9.982 ± 0.112	0.001
Lipid, %	0.094 ± 0.010	0.080 ± 0.015	0.446
Ash, %	0.740 ± 0.014	0.732 ± 0.012	0.674
Yolk Components			
Water, %	52.337 ± 0.422	51.801 ± 0.209	0.270
Protein, %	16.682 ± 0.193	16.526 ± 0.139	0.700
Lipid, %	27.232 ± 0.363	27.494 ± 0.176	0.523
Ash, %	1.943 ± 0.047	1.824 ± 0.024	0.037
Cholesterol, mg/g	11.900 ± 0.126	11.629 ± 0.135	0.157

The data are presented as means ± SEM (*n* = 12). For details on the *p*-value calculation procedures, please refer to Section 2 under Section 2.3.

**Table 4:** Albumen amino acid content (mg/g of egg albumen) following 30 weeks of HMB supplementation.

Item	Control	HMB	*p*-Value
Alanine	5.88 ± 0.08	5.74 ± 0.09	0.249
Arginine	5.63 ± 0.03	5.53 ± 0.08	0.250
Asparagine	10.70 ± 0.14	10.21 ± 0.12	0.012
Cysteine	3.84 ± 0.07	3.61 ± 0.07	0.028
Glutamic acid	13.71 ± 0.14	13.44 ± 0.18	0.312
Glycine	3.47 ± 0.04	3.35 ± 0.05	0.062
Histidine	2.28 ± 0.03	2.21 ± 0.03	0.137
Isoleucine	5.42 ± 0.12	5.79 ± 0.08	0.016
Leucine	8.65 ± 0.11	8.45 ± 0.13	0.623
Lysine	6.98 ± 0.10	6.66 ± 0.10	0.034
Methionine	4.73 ± 0.05	4.60 ± 0.08	0.174
Phenylalanine	5.93 ± 0.05	5.90 ± 0.10	0.840
Proline	3.28 ± 0.06	3.18 ± 0.07	0.311
Serine	7.16 ± 0.10	6.95 ± 0.08	0.134
Threonine	4.63 ± 0.06	4.65 ± 0.05	0.801
Tryptophan	4.88 ± 0.31	4.66 ± 0.42	0.707
Tyrosine	3.74 ± 0.02	3.71 ± 0.06	0.659
Valine	6.55 ± 0.09	6.35 ± 0.11	0.197

The data are presented as means ± SEM (*n* = 12). For details on the *p*-value calculation procedures, please refer to Section 2 under Section 2.3.

**Table 5:** Yolk amino acid content (mg/g of egg yolk) following 30 weeks of HMB supplementation.

Item	Control	HMB	*p*-Value
Alanine	7.94 ± 0.09	7.16 ± 0.36	0.053
Arginine	11.3 ± 0.12	10.95 ± 0.15	0.095
Asparagine	15.23 ± 0.21	13.70 ± 0.78	0.081
Cysteine	4.19 ± 0.12	4.02 ± 0.08	0.233
Glutamic acid	19.30 ± 0.23	18.86 ± 0.24	0.195
Glycine	4.73 ± 0.05	4.49 ± 0.04	0.003
Histidine	4.02 ± 0.03	3.69 ± 0.12	0.019
Isoleucine	7.91 ± 0.09	8.47 ± 0.08	<0.001
Leucine	13.87 ± 0.16	13.72 ± 0.14	0.481
Lysine	12.23 ± 0.10	12.44 ± 0.12	0.208
Methionine	6.14 ± 0.20	5.81 ± 0.37	0.440
Phenylalanine	6.76 ± 0.09	6.47 ± 0.15	0.118
Proline	6.05 ± 0.02	5.69 ± 0.10	0.004
Serine	13.23 ± 0.2	10.51 ± 0.81	0.006
Threonine	8.34 ± 0.08	8.46 ± 0.08	0.293
Tryptophan	6.62 ± 0.48	6.74 ± 0.57	0.875
Tyrosine	6.79 ± 0.06	6.73 ± 0.02	0.315
Valine	9.04 ± 0.08	8.02 ± 0.41	0.032

The data are presented as means ± SEM (*n* = 12). For details on the *p*-value calculation procedures, please refer to Section 2 under Section 2.3.

**Table 6:** Yolk fatty acid composition (% of total fatty acids) and health lipid indices following 30 weeks of HMB supplementation.

Item	Control	HMB	*p*-Value
C14:0	0.342 ± 0.010	0.346 ± 0.010	0.811
C16:0	24.429 ± 0.238	25.615 ± 0.315	0.007
C18:0	6.999 ± 0.097	7.143 ± 0.132	0.389
Σ SFA	31.770 ± 0.277	33.104 ± 0.267	0.002
C15:0	0.062 ± 0.006	0.063 ± 0.004	0.921
C17:0	0.181 ± 0.008	0.144 ± 0.005	0.001
Σ OCFA	0.243 ± 0.010	0.207 ± 0.007	0.014
C14:1c9	0.068 ± 0.006	0.086 ± 0.007	0.063
C15:1	0.097 ± 0.008	0.086 ± 0.006	0.299
C16:1c7	0.921 ± 0.041	0.876 ± 0.058	0.530
C16:1c9	2.909 ± 0.149	3.553 ± 0.173	0.010
C17:1c9	0.213 ± 0.006	0.203 ± 0.006	0.242
C18:1c9	44.632 ± 0.350	45.247 ± 0.364	0.236
C18:1c11	2.230 ± 0.057	2.557 ± 0.071	0.002
C20:1c11	0.227 ± 0.013	0.225 ± 0.010	0.862
Σ MUFA *cis*	51.297 ± 0.345	52.831 ± 0.271	0.002
C18:2n-6	11.718 ± 0.345	9.544 ± 0.249	<0.001
C18:3n-6	0.090 ± 0.007	0.078 ± 0.009	0.293
C20:2n-6	0.085 ± 0.009	0.068 ± 0.007	0.157
C20:3n-6	0.137 ± 0.012	0.112 ± 0.007	0.096
C20:4n-6	1.715 ± 0.043	1.657 ± 0.028	0.270
C22:4n-6	0.134 ± 0.005	0.120 ± 0.007	0.118
C22:5n-6	0.237 ± 0.022	0.291 ± 0.017	0.065
Σ PUFA n-6	14.117 ± 0.315	11.87 ± 0.238	<0.001
C18:3n-3	0.916 ± 0.045	0.574 ± 0.029	<0.001
C22:5n-3	0.148 ± 0.011	0.126 ± 0.008	0.125
C22:6n-3	1.464 ± 0.038	1.140 ± 0.030	<0.001
Σ PUFA n-3	2.528 ± 0.063	1.841 ± 0.040	<0.001
Σ PUFA	16.645 ± 0.301	13.711 ± 0.245	<0.001
Σ LC-PUFA	3.921 ± 0.070	3.515 ± 0.056	<0.001
Σ LC n-6	2.308 ± 0.055	2.248 ± 0.039	0.382
Σ LC n-3	1.612 ± 0.048	1.267 ± 0.031	<0.001
PUFA n-6/n-3	5.636 ± 0.219	6.480 ± 0.182	0.007
PUFA/SFA	0.525 ± 0.012	0.415 ± 0.009	<0.001
S/P	0.468 ± 0.006	0.498 ± 0.006	0.002
TI	0.786 ± 0.010	0.871 ± 0.010	<0.001
AI	1.547 ± 0.021	1.653 ± 0.024	0.003
h/H	2.468 ± 0.038	2.265 ± 0.037	0.001
NV	0.440 ± 0.007	0.474 ± 0.008	0.005

The data are presented as means ± SEM (*n* = 12). Only FA with a mean content of ≥0.05% is specified, but the total sum (Σ) of particular FA groups also includes FA < 0.05%. For details on the *p*-value calculation procedures, please refer to Section 2 under Section 2.3. SFA—saturated FA, OCFA—odd-chain FA, MUFA—monosaturated FA, PUFA—polyunsaturated FA, LC-PUFA—long chain PUFA, S/P—saturation index, TI—thrombogenic index, AI—atherogenicity index, h/H—hypocholesterolaemic/hypercholesterolaemic ratio, NV—nutritional value [43].

## Data Availability

The data presented in this study are available on request from the corresponding author.
